# Selection for Function in Early Life: Implications for Early‐Onset Pathologies

**DOI:** 10.1111/eva.70226

**Published:** 2026-04-10

**Authors:** Klara Asselin, Antoine M. Dujon, Jean‐Pascal Capp, Jean‐François Lemaître, Florence Pirard, Daniel Vaiman, Beata Ujvari, Pascal Pujol, James DeGregori, Aurora M. Nedelcu, Frédéric Thomas

**Affiliations:** ^1^ CREEC/CANECEV, MIVEGEC (CREES) Department, University of Montpellier, CNRS, IRD Montpellier France; ^2^ School of Life and Environmental Sciences Deakin University Waurn Ponds Victoria Australia; ^3^ Toulouse Biotechnology Institute University of Toulouse, INSA, CNRS, INRAE Toulouse France; ^4^ Université Lyon 1 CNRS, UMR5558, Laboratoire de Biométrie et Biologie Évolutive Villeurbanne France; ^5^ Institut Cochin U1016, INSERM, UMR8104 CNRS, Université de Paris Paris France; ^6^ Oncogenetic Department University Medical Centre of Montpellier Montpellier France; ^7^ Department of Biochemistry and Molecular Genetics University of Colorado Anschutz Medical Campus Aurora Colorado USA; ^8^ Department of Biology University of New Brunswick Fredericton New Brunswick Canada

## Abstract

Persistent pathological structures, such as tumors, fibrotic nodules, granulomas, microbial biofilms, or protein aggregates, are traditionally viewed as age‐related conditions that emerge after reproduction, when natural selection is less effective at eliminating traits expressed late in life. However, some pathologies with robust and organized architectures can arise surprisingly early, challenging this classical perspective. We recently proposed that intra‐organismal *selection for function*, a selective process operating within organisms and acting on non‐reproducing entities by favoring structural configurations that enhance stability, robustness, and novelty generation, may play a role in aging. Here, we suggest that this same process can also operate well before the so‐called selection shadow (i.e., life stages where natural selection is too weak to purge deleterious mutations). We identify three non‐mutually exclusive mechanisms that may promote this early‐life action: (i) initial local adaptive benefits, such as improved tissue repair or containment of infection; (ii) limited or context‐specific fitness costs, allowing structurally stable but abnormal configurations to persist undetected; and (iii) rapid environmental changes that reshape tissue‐level selective landscapes, driven by pollutants, endocrine disruptors, or novel diets. Recognizing early‐onset organized pathologies as by‐products of eco‐evolutionary tissue dynamics, rather than as mere developmental errors, reframes their biological significance and opens new therapeutic avenues. Instead of targeting cells exclusively, future strategies could focus on disrupting the functional architecture of pathological tissues and structures, offering novel means to prevent or control early‐life diseases shaped by internal selection forces.

## 1 Introduction

Persistent pathological structures characterized by internal organization and resilience (such as tumors, fibrotic nodules, microbial biofilms, or atherosclerotic plaques) are most commonly observed in older individuals and have therefore traditionally been interpreted as typical diseases of aging (Niccoli and Partridge 2012; Siegel et al. 2024; Montégut et al. 2024; Haikal and Weissert 2024; Omenn 2016; Andonian et al. 2024). Classical evolutionary theories assume that their persistence in populations, despite their negative effects on fitness, is facilitated by the decline in the strength of natural selection with age (i.e., the so‐called selection shadow (Hamilton 1966)). This decline is traditionally thought to allow the accumulation of mutations with age‐specific effects (Williams 1957). However, even in the absence of such genomic accumulation, the combined effects of waning somatic investment and increasing entropy may be sufficient to explain the exponential rise in damage and suboptimal gene expression observed in later life (Kirkwood 1977; Kirkwood and Rose 1997; Lemaître et al. 2024; Gems and de Magalhães 2021). Building on this logic, evolutionary genetic models, such as the antagonistic pleiotropy theory of aging (Williams 1957), have emphasized how trade‐offs between early‐ and late‐life fitness‐related traits contribute to aging, as well as its wide range of late‐onset pathologies (Kirkwood 1977; Austad and Hoffman 2018; Byars and Voskarides 2020; Mc Auley 2024; Nussey et al. 2013). Recent theoretical frameworks have further refined this view through the developmental theory of aging, suggesting that aging is partly a continuation of developmental programs selected for early‐life function that become hyper‐functional or detrimental later (Gems and Kern 2022; de Magalhães and Church 2005; Maklakov and Chapman 2019). Furthermore, recent syntheses link these developmental trade‐offs with mutation accumulation, showing how early‐life traits can degrade into pathology (Lemaître et al. 2024). Yet, mechanistically, these models generally consider pathology as the endpoint of passive processes: damage accumulation, repair failure, or unregulated cell proliferation.

Recently, an alternative but complementary perspective has been proposed using the concept of *selection for function* (Dujon et al. 2025). This concept recently proposed by Wong et al. (2023) is a more general and universal type of selection that can operate and shape the dynamics of both living and non‐living systems, as long as they meet these three requirements: “(1) they form from numerous components that have the potential to adopt combinatorially vast numbers of different configurations; (2) processes exist that generate numerous different configurations; and (3) configurations are preferentially selected based on function”. Unlike classical Darwinian evolution, which relies on genetic heritability across generations, each system subject to *selection for function* is shaped through the selection of advantageous configurations with respect to system‐level persistence. The three sources of selection that underpin “function” and drive the evolution of all systems are: static persistence (stability), dynamic persistence (robustness), and novelty generation of component part configurations (evolvability). To illustrate these principles, mineral evolution provides a useful example. Unlike biological organisms, minerals do not replicate or display heritable traits in the Darwinian sense. Yet Earth's mineralogy has diversified dramatically over geological time, from only a few dozen phases in the early Solar System to several thousand species today. This diversification can be understood in terms of changes in environmental conditions and thermodynamic stability: among the vast number of physically possible mineral configurations, only those that were stable under specific conditions persisted and accumulated over time. As environmental parameters such as pressure, temperature, and redox state shifted, new stable mineral species emerged and were retained, consistent with the notion of increasing functional information articulated in the mineral evolution framework (Hazen and Wong 2024). Using this framework, we suggest that certain pathological structures may persist not merely because of a lack of somatic maintenance or sub‐optimal gene expression in late‐life (Lemaître et al. 2024), but because local evolutionary dynamics within tissues actively favor stable and functional spatial configurations (Dujon et al. 2025). Within aging tissues shaped by inflammation, resource scarcity, or immune evasion, selection (within the tissue level) for resilient organizational architectures can emerge as by‐products, enabling pathological structures to persist and expand over time.

However, if *selection for function* within tissues contributes to the persistence of organized pathologies (i.e., pathological structures displaying a degree of internal organization and functional integration, such as tumors or atherosclerotic plaques), a major evolutionary expectation arises: such processes should be strongly opposed by natural selection at the organismal level. Indeed, these organized pathologies are expected to be repressed during early life, when natural selection is most intense and any trait compromising survival and/or reproduction is vigorously counter‐selected (Hamilton 1966; Medawar 1952). Paradoxically, despite this expectation, stable and organized pathologies are reported in young individuals, including during the early‐adulthood stage (Zhao et al. 2023; Lee et al. 2024; Dai et al. 2025; Arora et al. 2019). Similar phenomena may also occur in other vertebrates or complex multicellular organisms, suggesting a broader evolutionary principle (e.g., (Lyu et al. 2024)).

Here, we explore how *selection for function* within tissues can shape early‐onset organized pathologies. We identify the ecological and evolutionary mechanisms enabling the latter phenomenon, propose a conceptual framework integrating tissue dynamics and local selection pressures, and discuss the therapeutic implications of viewing early pathologies as eco‐evolutionary outcomes rather than mere developmental failures. *Selection for function* does not replace classical Darwinian evolution, but complements it by describing how functional organization can be selectively stabilized within biological systems.

## 2 The Paradox of Early‐Onset Organized Pathologies

Classical evolutionary theories predict that early life, particularly the reproductive phase, should be a period of strong purifying selection, rigorously eliminating traits or processes that compromise survival and/or reproduction (Hamilton 1966; Williams 1957; Kirkwood 1977; Austad and Hoffman 2018; Byars and Voskarides 2020; Mc Auley 2024; Nussey et al. 2013; Medawar 1952; Giaimo and Traulsen 2022; Kirkwood and Rose 1991; Li et al. 2023). Under this view, the emergence of persistent, organized pathological structures should be rare or strongly counter‐selected before the decline of natural selection forces associated with aging (Dujon et al. 2025). While this is generally true, a growing number of observations challenge this expectation. Pathologies such as endometriosis, granulomas (e.g., in tuberculosis or leprosy), microbial biofilms, benign tumors, and early tissue calcifications exhibit remarkable stability and internal organization, despite arising before or during the reproductive phase. Several of these conditions, such as endometriosis, can impair key fitness components, including fertility. In some cases, these structures can persist for years or even decades without being cleared by host mechanisms (Siegel et al. 2024; Dai et al. 2025; Deng et al. 2024; Lanphear et al. 1997; Hirsch et al. 2020; Maloney and Cohen 2023; Chen et al. 2022; Lou et al. 2023; Yu et al. 2020; Monfrini et al. 2023; Gui et al. 2024). However, many of these pathologies may fall under one or more exceptions that could attenuate purifying selection: (1) some are relatively rare and may escape selection due to low population prevalence; (2) others have emerged or become more prevalent in modern environments (e.g., due to endocrine disruptors, dietary shifts or medical progresses that drastically increased longevity in the case of the human species), creating evolutionary mismatches; (3) and some are linked to extrinsic causes such as chronic pathogens, where host‐level selection acts on containment rather than elimination. Clarifying how each pathology aligns with these exceptions helps understand why functionally persistent but potentially costly structures can nonetheless arise and persist in early life.

This raises a fundamental evolutionary paradox: how can processes favoring the stabilization and persistence of pathological structures operate at the tissue level during a life stage when most reductions in organismal fitness should, in principle, be strongly selected against? While purifying selection is indeed intense in early life, several factors may attenuate its efficiency. Some weakly deleterious traits, particularly those with fitness costs below the drift barrier (s < 1/Ne, meaning their selective disadvantage is too small to be efficiently eliminated by natural selection), may escape purging. In humans, for example, traits with fitness effects smaller than ~0.0001 could persist despite being slightly deleterious. In addition, ecological mismatches, extrinsic drivers, and tissue‐level selective dynamics can further favor the persistence of such organized pathologies. Understanding this paradox therefore requires rethinking the interplay between drift, local selective landscapes, and the evolutionary logic of pathogenesis.

## 3 Mechanisms Enabling Intra‐Organismal *Selection for Function* in Early Life

### 3.1 Early‐Life Benefits of Tissue Organization

Certain pathological structures may be the by‐product of physiological processes that confer clear adaptive advantages in early life. Rapid tissue repair following injury, leading to scarring, local containment of pathogens, or structural reinforcement of vulnerable tissues can create microenvironments that favor the stabilization of specific spatial configurations that become new unconventional niches inside the body.

They seem to be initiated by the tissue's ability to adapt to disturbance. While this capacity is necessary to maintain the reproductive capacity of the whole organism during early life, the return to the initial tissue structure and microenvironment is not always the outcome. In these newly formed structures, a novel dynamic equilibrium that confers the basic ground of *selection for function* is found, either purely developmental when derived solely from host cells (e.g., endometriosis, benign tumors), or mixed when composed of microbial cells interactions with host cells (e.g., some granulomas, biofilms). Initially, such configurations promote short‐term survival, resilience, or protection against immediate threats, aligning with selective pressures for host fitness. However, once established, these organized structures may become excessively stabilized or maladaptively persistent under changing conditions. For instance, fibrotic nodules formed as part of wound healing responses can, over time, impair tissue function if remodeling fails (Wynn 2008; Ku et al. 2024; Di et al. 2025; Distler et al. 2019). This echoes the view that healing itself is an evolved, functionally optimized process, which can produce pathological outcomes such as fibrosis, when regulation is lost or context changes (Matsumoto‐Oda et al. 2025). Similarly, granulomas are organized aggregates of immune cells that typically form in response to chronic infections, such as tuberculosis or leprosy. Yet, these structures can also arise in autoimmune, toxic, or allergic conditions and persist for decades. While they may sometimes evolve into pathological masses that damage surrounding tissues (Lyu et al. 2024; Weeratunga et al. 2024; Nakamizo and Kabashima 2025; Serrano‐Coll et al. 2024), this can also be viewed as a standard evolutionary trade‐off: the long‐term impairment of tissue functionality may be tolerated as a cost for ensuring pathogen containment and host survival in the short term. These examples illustrate that early‐life benefits at the tissue level can inadvertently set the stage for long‐term pathogenesis. In such cases, the initial selective advantage at the organismal level may gradually transition into local *selection for function* at the level of the pathological structure itself, reshaping the tissue microenvironment in ways that promote persistence rather than resolution.

### 3.2 Limited or Context‐Dependent Fitness Costs

Among the local tissue configurations that are stable and pathological, not all have dramatic effects on growth and reproductive success. Some structures may arise in non‐essential tissues, remain asymptomatic for long periods, or affect only specific sexes, tissues, or age classes. In such cases, their presence may escape strong purifying selection, allowing them to persist and even stabilize without immediate fitness penalties. For example, the ectopic growth of endometrial tissue characterizing endometriosis is associated with strongly impaired fertility in some women, but in many cases remains undiagnosed or produces mild, non‐debilitating symptoms throughout the reproductive phase (Miranda et al. 2024; Emami 2025; Kimber‐Trojnar et al. 2021; Strawn 2025; Rawson 1991). The initiation of endometriosis might be explained by deleterious alleles persisting below the drift barrier due to context‐dependent effects on fertility, as suggested by mutation accumulation models (Lemaître et al. 2024). However, intra‐organismal *selection for function* explains the subsequent step: how these endometriosis lesions organize into stable structures (see also Box 1). Once the tissue is established outside the uterus, local *selection for function* would favor configurations that can remodel the extracellular matrix and evade immune surveillance, ensuring the physical persistence of the lesion regardless of its initial genetic cause. Similarly, benign tumors such as lipomas or localized calcifications in soft tissues often impose negligible physiological costs during early life, becoming problematic only later or under specific environmental, physiological stresses (Boutry et al. 2022; Bhandari 2021; Derin and Yaprak 2017; Vidavsky et al. 2021; Masroori et al. 2025). This context‐dependence of fitness costs creates ecological niches within the organism where tissue‐level *selection for function* can operate relatively unchecked. Apart from contexts involving infectious agents, such phenomena might be confined to tissues or locations that have developed less stringent barriers against cell growth, where the fitness costs of abnormal tissue structuration are inherently lower. Initially benign or neutral configurations may progressively reinforce their internal organization, especially if local microenvironments or tissue dynamics favor their persistence over clearance.

### 3.3 Novel Ecological Contexts Created by Modern Environments

Contemporary human environments differ profoundly from those in which most of our evolutionary history unfolded (Corbett et al. 2018; Manus 2018). Exposure to pollutants, endocrine disruptors, novel pathogens, altered diets, sedentary lifestyles, and chronic low‐grade inflammation (“inflammaging”) have reshaped the microenvironment within tissues in ways that were historically rare or absent. At the same time, advances in medicine and reduced undernutrition have prolonged lifespan, extending the period during which *selection for function* can act. These rapid ecological shifts have created physiological mismatches between evolved biological systems and the novel conditions of modern life, thereby promoting tissue dysfunction and novel local selective pressure that can favor the emergence of persistent pathological structures. It is important to note that in such rapidly shifting environments, *selection for function* may favor robust pathological structures with high dynamic persistence: those capable of maintaining their integrity despite variable stress signals. For instance, environmental pollutants and endocrine disruptors have been shown to interfere with immune regulation, metabolic homeostasis, and cellular signaling, contributing to chronic diseases such as obesity, diabetes, and cancer (Kabir et al. 2015; Sørensen et al. 2003; Vrijheid et al. 2016). Based on liver carcinogenesis, Farber and Rubin suggested 35 years ago that pre‐cancerous steps are underlined by an early phenomenon of cellular adaptation to carcinogens' exposure (Farber and Rubin 1991). More general, such “clonal adaptation” to novel ecological contexts might pave the way for organized abnormal tissue structures submitted to *selection for function*, that remain non‐invasive but develop according to their own evolutionary trajectory. Moreover, endocrine disruptors are an example of such environmental factors, now acknowledged to cause developmental and neoplastic issues where *selection for function* can occur (Schug et al. 2016; Colborn et al. 1993). Likewise, modern diets and sedentary behavior alter gut microbiota composition and inflammatory tone, facilitating low‐grade systemic inflammation and premature tissue degradation (Gluckman and Hanson 2006). The phenomenon of “inflammaging” reflects a systemic drift in tissue microecology that can favor the early emergence of stable, functionally organized pathologies (Fülöp et al. 2019; Franceschi et al. 2007), especially when immune cells are interacting with microbes (e.g., infection‐associated granulomas, biofilms). Together, these anthropogenic pressures reconfigure local selective landscapes, potentially enabling the early action of *selection for function* at the tissues' level, in contexts previously constrained by strong purifying selection at the organismal level. These changes can prematurely degrade tissue integrity, modify immune responses, or alter resource availability at the local level, effectively shifting the selective landscape within tissues. Under such altered conditions, configurations that would have been transient or maladaptive in ancestral environments may gain a selective advantage, stabilizing into persistent pathological structures even during early life. For instance, chronic exposure to environmental toxins can induce persistent fibrotic responses in organs such as the liver or lungs; high‐sugar or high‐fat diets can promote early calcifications and microvascular changes; and endocrine disruptors may interfere with normal tissue organization in reproductive systems. Together, these novel ecological pressures create unprecedented opportunities for within‐organism *selection for function* to operate outside its historically expected temporal window, challenging classical predictions about the timing and emergence of organized pathologies (Andonian et al. 2024; Kabir et al. 2015; Sørensen et al. 2003; Vrijheid et al. 2016; Gluckman and Hanson 2006; Bonnet and Cheval 2023; McMichael 2001; Bălă et al. 2021; Macedo et al. 2023; González Olmo et al. 2021).

## 4 Toward an Eco‐Evolutionary Framework for Tissue Pathology

Recognizing tissues as dynamic ecosystems subjected to local selective pressures provides a powerful conceptual shift for understanding early‐onset pathologies. Rather than viewing persistent pathological structures as the mere consequences of developmental errors or genetic anomalies, this perspective emphasizes their emergence as potential products of localized eco‐evolutionary dynamics. Within tissues, cells interact not only with one another but also with their microenvironment, creating a complex web of ecological interactions shaped by gradients of oxygen, nutrients, mechanical forces, immune signals and extracellular matrix (ECM) properties. When this microenvironment is altered (by injury, infection, environmental toxins, or metabolic stress), the local selective landscape can shift dramatically, favoring configurations that enhance persistence and internal organization (Malta et al. 2021; Conrad et al. 2013; Zahir et al. 2020; Kobayashi et al. 2024; Le et al. 2021; Dinsdale et al. 2021; Sarsenova 2025; Ibrahim‐Hashim et al. 2017; Rampias 2020; Janiszewska 2020; Huang et al. 2025). In healthy tissues, cell fate is tightly regulated by higher‐level control mechanisms that suppress lower‐level selection. However, tissue control is not absolute; it operates within ecological and physiological constraints. When local perturbations generate alternative structural configurations (e.g., during chronic inflammation or repeated repair), regulatory systems may stabilize a new local equilibrium rather than restoring the original architecture. In such contexts, selection for function does not require full escape from tissue control, but instead reflects a shift in the regulatory landscape in which certain configurations exhibit greater persistence or robustness within the altered microenvironment. In this framework, pathologies such as granulomas, microbial biofilms, fibrotic nodules, benign tumors, and early calcifications can be interpreted as emergent structures stabilized by *selection for function* within their local tissue ecosystems. Each represents a case where functional organization at a lower‐level increases system persistence, even if it ultimately compromises higher‐level organismal fitness. Viewing pathological tissues as eco‐evolutionary systems thus unifies a wide range of early‐ and late‐onset diseases (Dujon et al. 2025) under a common evolutionary logic. It also highlights novel therapeutic opportunities aimed not only at eliminating pathological cells, but also at disrupting the ecological and structural conditions that sustain pathological organization.

To test the role of selection for function in early‐onset pathologies, we propose several empirical approaches. If these pathologies are shaped by intra‐organismal *selection for function* rather than solely by genetic determinism or passive degeneration, this framework generates specific, falsifiable predictions. First, independent lesions subjected to similar ecological constraints should exhibit convergence toward comparable structural and functional architectures, even when initiated by distinct molecular events. Second, during early lesion development, configurational diversity should become increasingly constrained over time, with only those spatial organizations that enhance stability, robustness, or persistence being preferentially retained. This would reflect functional filtering rather than random drift. Third, experimentally disrupting the architectural or ecological coherence of a lesion should destabilize its persistence, even if the initiating genetic alterations remain intact. These predictions can be tested using organoid systems, longitudinal in vivo imaging, controlled microenvironmental perturbations, and targeted disruption of extracellular matrix organization or intercellular communication networks. Demonstrating convergence, configurational filtering, and architecture‐dependent persistence would provide empirical support for selection for function operating within tissues.

## 5 Implications for Therapeutic Strategies

Recognizing the role of intra‐organismal *selection for function* in shaping early‐life pathologies calls for a reconsideration of therapeutic strategies. Traditional interventions, primarily focused on eliminating pathological cells, may overlook the deeper organizational resilience that sustains persistent pathological structures. To be effective, treatments should target not only individual cells but also the functional architectures and ecological conditions that enable pathological persistence (Greaves and Maley 2012).

Several complementary strategies emerge from this perspective, notably disrupting the structural integrity of pathological entities such as fibrotic nodules, calcifications, or invasive architectures like endometriotic lesions by targeting the ECM (Tomos et al. 2025; Zhang, Al‐Danakh, et al. 2025; Rossi et al. 2025; Zhang, Zhou, and Kong 2025). For example, anti‐fibrotic agents such as pirfenidone and nintedanib, currently used in the treatment of idiopathic pulmonary fibrosis, do not target specific cell types. Instead, these molecules interfere with ECM remodeling and fibroblast‐driven signaling loops that sustain fibrotic tissue architecture and promote its persistence (Richeldi et al. 2014; King et al. 2014). Another strategy involves modulating local microenvironmental conditions, as pathological structures thrive within specific niches characterized by altered pH, oxygen gradients, nutrient distributions, or immune signaling. Therapeutic modulation of these local parameters could erode the within‐tissue selective advantages that stabilize pathological organizations (Shah et al. 2025; Pandit and Yurdagul 2025). Additionally, interfering with intercellular communication networks is a promising approach, as stable pathological structures often rely on intricate signaling among their constituent cells (Di et al. 2025; Bell and Muniyan 2025; O'Riordan et al. 2025; Chigozie et al. 2025; Okamoto et al. 2025; Razzaq Meo et al. 2025; Lasse‐Opsahl et al. 2025). In the context of bacterial biofilms, quorum sensing inhibitors such as synthetic furanones or subinhibitory doses of azithromycin aim to disrupt the coordinated communication networks that stabilize these complex microbial structures. This intercellular communication interference weakens the ecological and architectural cohesion of the biofilm, without necessarily eradicating the bacteria (Hentzer and Givskov 2003; Rasmussen and Givskov 2006). Lastly, reducing persistent inflammatory signals and minimizing exposure to environmental risk factors could prevent the emergence or reinforcement of pathological structures, particularly in early life when tissues are more plastic (Andonian et al. 2024; Kabir et al. 2015; Sørensen et al. 2003; Vrijheid et al. 2016; Gluckman and Hanson 2006; Fülöp et al. 2019; Bonnet and Cheval 2023; McMichael 2001; Bălă et al. 2021; Macedo et al. 2023; González Olmo et al. 2021; Darbre 2021; Pironti et al. 2021; Wild 2012). However, modulating local microenvironmental conditions to disrupt a specific pathology carries risks. This approach could inadvertently create a new empty niche or selective pressure favoring a different type of pathological organization. For instance, suppressing the immune response to reduce granuloma formation might open the door to uncontained infection. Therefore, therapies targeting *selection for function* must be designed with a full understanding of the tissue ecosystem to avoid replacing one stable pathology with another.

These cases illustrate how targeting the structural and ecological coherence of pathological systems can offer therapeutic leverage, in line with the principle of counteracting *selection for function* within tissues.

A summary of early‐onset pathologies shaped by intra‐organismal *selection for function*, along with their main mechanisms and therapeutic targets, is provided in Tables 1A and 1B below. While Table 1A summarizes the eco‐evolutionary mechanisms and potential therapeutic strategies for early‐onset organized pathologies, Table 1B evaluates to what extent each pathology type fulfills the three formal criteria proposed by Wong et al. for *selection for function* to operate (Wong et al. 2023). Importantly, the targets listed do not focus on individual cells, but on destabilizing the organizational structures that maintain pathological persistence.

**TABLE 1A:** Early‐onset pathologies potentially shaped by intra‐organismal *selection for function*: associated mechanisms and therapeutic targets.

Pathology type	Mechanism favoring *selection for function*	Potential therapeutic target
Endometriosis	Hormonal modulation, tissue repair benefits	Destabilizing invasive architecture
Granulomas (TB, Leprosy)	Infection containment	Disrupting immune cell organization
Microbial biofilms	Resistance to host immunity and antibiotics	Targeting extracellular matrix and communication signals
Benign tumors/nodules	Local tissue reinforcement	Modulating extracellular matrix and tissue dynamics
Calcifications/crystals	Stabilization in specific microenvironments	Preventing mineral aggregation

*Note:* This table summarizes representative early‐onset pathological structures that exhibit internal organization and persistence. For each pathology type, we outline the main mechanisms likely to promote intra‐organismal *selection for function*, such as initial adaptive benefits, context‐dependent fitness costs, or ecological novelty. We suggest therapeutic strategies that target not only individual cells, but also the structural or ecological properties sustaining the pathological configuration.

**TABLE 1B:** Evaluation of early‐onset pathologies against Wong et al.'s three criteria for *selection for function*.

Pathology	Condition 1 (compositionality)	Condition 2 (generativity)	Condition 3 (functional filtering)
Endometriosis	Epithelial, stromal and immune cells	Hormonal cycling, tissue remodeling	Lesions resist clearance, maintain local function
Granulomas	Immune cell aggregates (macrophages, T‐cells, etc.)	Chronic immune activation, recruitment waves	Contain persistent pathogens for decades
Biofilms	Bacterial populations and ECM	Mutation, quorum sensing, adhesion variability	Increased resistance to antibiotics/immune attack
Benign tumors	Clonal cells and ECM	Cell proliferation, stromal remodeling	Persist with minimal host damage
Calcifications	Mineral aggregates and matrix proteins	Crystallization, ECM remodeling	Stabilize local microenvironments

*Note:* According to Wong et al. (2023), *selection for function* can operate in any system that fulfills the following three conditions: (1) it is composed of multiple interacting components capable of generating a wide range of configurations (compositionality), (2) it involves processes that produce diverse configurations over time (generativity), and (3) some configurations are preferentially retained based on functional performance (functional filtering). This table evaluates whether the early‐onset pathologies listed in Table 1A meet these criteria, thereby supporting their interpretation as products of intra‐organismal *selection for function*.

Abbreviation: ECM, extracellular matrix.

## 6 Conclusion

*Selection for function* affecting levels below the organism (including non‐reproducing entities) represents a powerful and underappreciated evolutionary force, capable of shaping organized pathological structures not only during aging (Dujon et al. 2025), but also surprisingly during the developmental period and early adulthood. By revealing how stable pathologies can emerge under strong selective constraints within tissues, this framework challenges classical views of disease origins. Traditionally, such structures have been attributed solely to developmental errors or age‐related decline in organismal functioning at different levels of biological organization. Our perspective highlights that early‐onset pathologies, from endometriosis to granulomas, biofilms, and benign tumors, may arise from localized eco‐evolutionary dynamics within tissues, where initial adaptive benefits, limited fitness costs, or novel environmental pressures allow *selection for function* to operate. This perspective unifies a broad range of seemingly disparate pathologies under a common evolutionary logic. It emphasizes the need to view tissues not as passive substrates, but as dynamic ecosystems subject to internal selective forces affecting both reproducing (cells) and non‐reproducing (tumors, granulomas, biofilms) entities that favor their own persistence and robustness; hence, maybe explaining the difficulty of efficient medical interventions against these diseases. Recognizing these dynamics has profound therapeutic implications. Rather than focusing exclusively on the elimination of pathological cells, future strategies could aim at destabilizing the functional architectures and microenvironmental conditions that sustain pathological persistence, opening new avenues for preventive and curative interventions. Exploring the prevalence, mechanisms, and vulnerabilities of intra‐organismal *selection for function* in early life, across species and biological contexts, represents a fertile field for future research. By integrating ecological, evolutionary, and biomedical insights, we may uncover novel strategies to intercept the earliest stages of organized pathology, long before their consequences become irreversible.

BOX 1 **Endometriosis as an example of *selection for function*.**

According to Wong et al., *selection for function* can operate in any system that fulfills the following three conditions: (1) it is composed of multiple interacting components capable of generating a wide range of configurations (compositionality), (2) it involves processes that produce diverse configurations over time (generativity), and (3) some configurations are preferentially retained based on functional performance (functional filtering) (Wong et al. 2023). This box details how endometriosis meets these criteria.

**Condition 1 (compositionality)**.

An endometriotic lesion is composed of several interacting cell types, including endometrial‐like epithelial and stromal cells, as well as immune cells present in the peritoneal environment. There is a high degree of immune cell diversity, including macrophages, NK cells (Natural Killer), regulatory T cells, and neutrophils. Nerve fibers and extracellular matrix components are also part of this complex architecture. This cellular heterogeneity provides a combinatorially rich system capable of adopting multiple spatial and functional configurations, thereby establishing the structural preconditions for configurational variation (As‐Sanie et al. 2025; Mariadas et al. 2025; Huang et al. 2023).

**Condition 2 (generativity)**.

The lesion exhibits active biological processes that continuously generate variation in local states. Persistent inflammatory signaling, altered cytokine profiles (e.g., sustained IL‐1β and TNF‐α expression), and dysregulated estradiol‐driven proliferation together with reduced progesterone responsiveness contribute to fluctuating microenvironmental conditions. These processes generate multiple potential configurations of immune–stromal interactions and extracellular matrix organization (As‐Sanie et al. 2025; Mariadas et al. 2025; Huang et al. 2023).

**Condition 3 (functional filtering)**.

Among the range of generated configurations, those that enhance lesion stability and persistence are preferentially retained. For example, remodeling of the extracellular matrix through enzymes such as MMP‐2 and MMP‐9 reinforces structural anchoring. Such configurations promote lesion persistence, even when associated with negative organism‐level fitness consequences such as pain or reduced fertility. In this sense, the lesion represents a configuration that has been stabilized through functional filtering at the tissue level (As‐Sanie et al. 2025; Mariadas et al. 2025; Huang et al. 2023).

## Funding

This work was funded by the CNRS (IRP CANECEV), the HOFFMANN Family, and by the following grant: EVOSEXCAN project (ANR‐23‐ce13‐0007).

## Conflicts of Interest

The authors declare no conflicts of interest.

## Data Availability

Data sharing is not applicable to this article as no datasets were generated or analysed during the current study.
